# Myocardial fibrosis in athletes: Additional considerations

**DOI:** 10.1002/clc.23466

**Published:** 2020-09-24

**Authors:** Łukasz A. Małek, Chiara Bucciarelli‐Ducci

**Affiliations:** ^1^ Department of Epidemiology Cardiovascular Disease Prevention and Health Promotion, National Institute of Cardiology Warsaw Poland; ^2^ MSc in Sports Cardiology St. George's University of London London UK; ^3^ Bristol Heart Institute, Bristol National Institute of Health Research (NIHR) Biomedical Research Centre University Hospitals Bristol NHS Trust and University of Bristol Bristol UK

Dear Editor,

We would like to thank Martin et al^1^ for their interest with our recent review on the current perspective in myocardial fibrosis in athletes and the provided comments.^2^ In their letter, the authors suggest that hypertrabeculation should be included as another expression of the athlete's heart after careful differentiation from left ventricular noncompaction (LVNC).^3^ Furthermore, they point out on *isolated arrhythmogenic left ventricular dysplasia*, which, as they postulate, should be included separately in the differentiation between normal and abnormal imaging findings in athletes.

Our review was mainly focused on the summary and discussion of most common patterns of fibrosis observed in asymptomatic athletes described in the observational studies, including nonischemic insertion‐point fibrosis or small mid‐wall/subepicardial fibrosis and small ischemic‐type fibrosis (mostly in veteran athletes). Those patterns are usually found in athletes with otherwise normal hearts or presenting features of an athlete's heart defined as a physiological adaptation to physical exercise including balanced enlargement of heart chambers with or without mild left ventricular hypertrophy.^4^ Enlarged ventricles lead to the extension of existing trabeculae, which may appear as hypertrabeculation, but as there is not true structural change to the heart, these feature is not typically considered as a part of an athlete's heart. Apart from lack of symptoms, athletes with incidentally found small fibrosis usually have normal electrocardiogram (ECG), lack of significant arrhythmia and normal physical capacity.

In the last part of our review, we present how fibrosis detection and other cardiac magnetic resonance (CMR) markers can be used to distinguish between athlete's heart and pathology including arrhythmogenic right ventricular hypertrophy (ARVC). ARVC may include the cases of *isolated arrhythmogenic left ventricular dysplasia* and for this reason, it is recently referred to as simply arrhythmogenic cardiomyopathy.^4^ These athletes may also have nonischemic patterns of fibrosis in the left ventricle as pointed out in the review. However, they are usually accompanied by other CMR features atypical for the athlete's heart. Athletes suspected of cardiomyopathy are also more likely to present symptoms, to have changes on resting ECG, accompanying arrhythmia and/or decreased physical performance. All this information should be taken into account in the assessment of athletes suspected of pathology, with CMR being only one of the supporting imaging methods in that process.

## CONFLICT OF INTEREST

The authors declare no potential conflict of interest.
